# Atypical hemolytic uremic syndrome: Unique clinical presentation linked to rare *CFHR5* mutation

**DOI:** 10.1002/jha2.288

**Published:** 2021-09-14

**Authors:** Sofia Menotti, Martino Donini, Giuseppina Pessolano, Livia Tiro, Maurizio Cantini, Jacopo Croce, Matteo Morandi, Filippo Mazzi, Katia Donadello, Oliviero Olivieri, Francesco Dima, Sergio De Marchi, Giovanni Gambaro, Enrico Polati, Lucia De Franceschi

**Affiliations:** ^1^ Department of Medicine University of Verona and AOUI Verona Verona Italy; ^2^ Department of Transfusion Medicine University Hospital Verona Italy; ^3^ Department of Neuroscience, Biomedicine and Movement, Section of Clinical Biochemistry University of Verona and AOUI Verona Italy; ^4^ Department of Surgery, Dentistry, Paediatrics and Gynaecology University of Verona & AOUI Verona Italy

**Keywords:** complement, eculizumab, microangiopathy, plasmapheresis

**To The Editor,**

In adults, the diagnosis and the clinical management of atypical hemolytic uremic syndrome is still a challenge for hematologists [1, 2, 3]. Here, we report a case of a 40‐year‐old previously healthy man presented to the emergency department with fever (39.6°C) and bilateral persistent foot pain. He has been 2 days earlier by his primary care doctor due to lower extremities pain and a sensation of low body temperature during physical activity. At admission, he was found diaphoretic, apyretic (reported paracetamol intake at home), tachycardic, with marbled lower limbs and lack of sensitivity in both feet. The patient's history was negative for recurrent infections, kidney diseases, immune‐rheumatological, or cardiovascular disorders. He smoked 20 cigarettes/day.

Chest X‐ray showed a small pleural effusion in the lower left lobe, and ECG sinus tachycardia. Several blood test could not be performed due to marker hemolysis. Arterial blood gases were indicative of compensated metabolic acidosis (Table 1; Figure 1A). Therapy consisted of hydration, in conjunction with antibiotic and antifungal therapy (clarithromycin, piperacilline‐tazobactam and isavuconazole). A CBC showed normal Hb values with neutrophilic leukocytosis and thrombocytopenia (Hb 14.3 g/dl, WBC 18,920/μl, PLTs 26,000/μl). This was associated with increased total bilirubin 2.4 mg/dl, LDH and reduced haptoglobin (Table 1, Figure 1A). An elevated serum creatinine (3.96 mg/dl) was accompanied by biochemical signs of inflammation (CRP: 127 mg/L, PCT: 64.8 ng/ml) and coagulopathy (Table 1; Figure 1A). Myoglobin 224 ug/L and CK 231 U/L were at increased to upper normal values. Blood smear revelated anysopoikylocytosis and schistocytes (1‐2/field). Acute, unexpected hemolytic anemia, thrombocytopenia, and altered coagulation indices may suggest intravascular disseminated coagulation (DIC) or a thrombotic microangiopathy. These conditions are not mutually exclusive, since DIC and TMA can be simultaneously present and are of difficult distinction in critically ill patients [4, 5, 6]. Twelve hours after admission, the patient was tachycardic (135 beats/min) but stable, hemodynamically. He showed a severe compensated lactic metabolic acidosis with hypocapnia with tachypnea, patient became anuric despite intravenous fluid therapy and use of diuretics. A Retiform purpura appeared on the lower limbs, while one with confluent petechiae appeared on the upper limbs and face. Dysaesthesia and paraesthesia were reported in the lower limbs (distal feet and third leg) and hands (Figure 1A,B). A chest CT showed bilateral pleural effusion with a left > right bi‐basal compressive atelectasis, and ground glass lesions bilaterally in the medium and superior fields; an abdomen CT showed swollen kidneys bilaterally with no vascularization of the cortical area and enhancement within the limits of the norm in the medullary area (Figure 1C).

**TABLE 1:** Hematological and biochemical data

	T0	3 months	6 months	1 year	*Normal range*
Hb (g/dl)	11.7	12.2	8.4	11.3	*13.5–17.5*
Reticulocytes (*10^9/μl)	145.3	38.5	76.4	62.6	*≤60.00*
WBC (*10^3/μl)	28.8	10.83	10.68	8.83	*4.5–11.0*
PLT (*10^3/μl)	26	432	456	338	*150–400*
Creatinine (mg/dl)	3.96	4.15	3.06	2.36	*0.59–1.29*
Total bilirubin (mg/dl)	2.4	0.43	0.19	NA	*<1.05*
LDH (U/L)	1365	219	NA	179	*135–225*
CRP (mg/L)	127	7	126	NA	*<5*
Pro‐calcitonin (ng/ml)	64.8	0.11	NA	NA	*<0.5*
PT (sec)	4.27	NA	1.02	0.92	*0.8–1.2*
aPTT (sec)	5.16	1	1.02	NA	*0.8–1.2*
Fibrinogen (g/L)	<0.35	3.85	6.36	NA	*2–4*
D‐dimer (ug/L)	>10000	NA	NA	NA	*<500*
Creatine kinase (U/L)	452	44	NA	NA	*40–300*
ADAMTS 13 (%)	24	NA	NA	NA	*60–130*
Haptoglobin (g/L)	<0.08	NA	NA	NA	*0.3–2*
C3 (g/L)	0.69	1.08	NA	NA	*0.9–1.8*
C4 (g/L)	0.18	0.45	NA	NA	*0.1–0.4*
C5a* (ng/ml)	32	NA	NA	NA	*1–5*

Abbreviations: aPTT, activated partial thromboplastin time; BMI, body max index; eGFR, estimated glomerula filtration rate; Hb, hemoglobin; PLTs, platelets; PT, prothrombin time; TBil, total bilirubin; WBC, white blood cells.

*C5a was determined in our research laboratory (EIA Quidel Corp., San Diego, CA; USA).

T0: time 0, corresponding to patient hospital admission.

**FIGURE 1:**
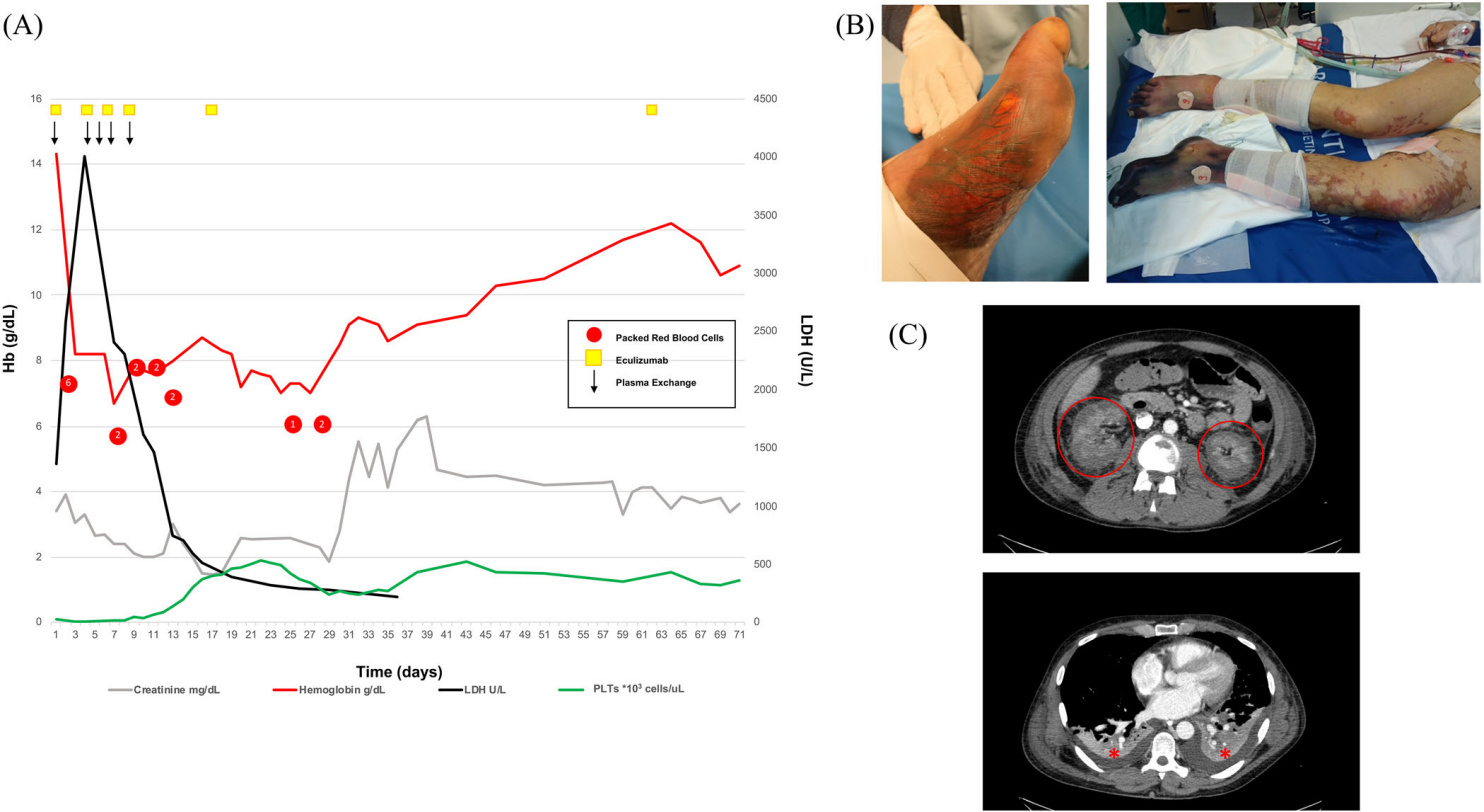
(**A)** Summary of patient clinical course. Hemoglobin (Hb, g/dl) red line; creatinine (mg/dl) gray line; lactate dehydrogenase (LDH, U/L) black line; platelets (PLTs, cell*10^3^ cells/μl) green line. Red points correspond to transfusion of packed red blood cells; yellow squares: eculizumab administration; black arrow: plasma exchange (PEX). (**B)** Severe microangiopathy involving soft tissue left foot (3rd day, **left panel**). Ischemic left and right lower limb and bilateral purpura (8th day, **right panel**). **(C)** Abdominal CT shows acute kidney sufferance **(upper panel,** red circle); chest CT shows bilateral small pleural effusions (**lower panel,** red asterisks)

Repeated CBC and biochemical studies showed severe hemolytic anemia, and thrombocytopenia associated with increased LDH.

Few schistocytes (1‐2/field) were present at the peripheral blood smear. ADAMTS13 activity was reduced to 24.3% (Table 1). Plasma C3 was decreased with normal C4 and increased C5a (Table 1) [7]. Shiga‐toxin, stool cultures, blood cultures, pharyngeal swab for H1N1 and respiratory viruses (rhinovirus and metapneumovirus) were negative. The antibiotic spectrum was expanded by introducing daptomycin due to the appearance of right basal pneumonia and the persistence of increased inflammatory markers. *Streptococcus pneumoniae* was isolated only in one blood culture. The reduction of C3 associated with normal activity of ADAMTS13 and increased C5a values in a patient with acute severe microangiopathic hemolytic anemia, thrombocytopenia and renal failure is suggestive of complement‐mediated aHUS. Plasmapheresis (PEX) was initiated in combination with eculizumab, a monoclonal antibody directed against the C5b9 (Figure 1A; Table 1). Continuous renal replacement therapy (CRRT) was also introduced because of persistent anuria. An OXIRIS filter (Baxter) was used to remove cytokines. The patient was intubated because of the deterioration of the gas exchange and transfused with both packed red cells and platelets (Figure 1A). A total of four PEX (on day 4, 5, 6, and 8) were performed, and eculizumab was administered on day 4 (300 mg adjusted dose after PEX), day 6 (900 mg), day 8 (300 mg), day 16 (900 mg), and day 28 (1200 mg; Figure 1A). Vaccines for ACWY Meningococcus (day 6) and B meningococcus (day 15) were administered. Clarithromycin and daptomycin were discontinued on day 5 because of the identification of *Staphylococcus epidermidis* in the blood culture. On day 9, a relapse of inflammatory markers such as CRP and PCT was observed. MRSA *Enterococcus faecalis* was isolated in the blood cultures, and daptomycin was reintroduced for 3 additional days. Immunological tests such as ANA, ENA, ANCA, anti‐cardiolipin immunoglobulin G (IgG), and anti‐cardiolipin b2‐glycoprotein I complex antibody were negative. NGS genomic analysis for 15 genes associated with TMA/HUS was performed. The rare missense variant c1541T → G on *CFHR5* gene in heterozygote state was identified. Renal function slowly improved, allowing a transition from continuous dialysis (CRRT) to biweekly hemodialysis combined with furosemide stimulation. Normalization of the patient's neurologic state was observed on day 14. A gradual improvement of the skin lesions was observed with complete recovery on day 15 after hospital admission. However, both lower limbs were severely compromised due to a massive thrombosis of distal microcirculation and dry necrosis involving toes and soles of both feet developed (Figure 1C). Early diagnosis of aHUS with PEX and eculizumab therapies resulted in a rapid hematologic remission, resolution of neurologic symptoms and improvement of the renal function as typically observed [5, 8]. Indeed, studies in patients with complement‐mediated HUS have shown that eculizumab reduces both mortality and acquired end‐stage kidney disease [9, 10, 11].

Four to five months after hospital discharge, patient had recovered to a G4 chronic kidney disease stage (creatinine 3.5 mg/dl, eGFR 20 ml/min/1.73m^2^, K^+^ 5.4 mmol/L), and hemodialysis was discontinued. Bilateral below knee amputations were carried out. Eculizumab (900 mg) is still being administered every 4 weeks without clinical or laboratory signs of relapse.

Our case describes an aHUS with superimposed DIC with a rare missense variant c1541T → G on *CFHR5* and catastrophic clinical presentation. Sakurai et al have recently analyzed a case series of aHUS, secondary TMA, and DIC [2]. They concluded that the diagnosis of DIC *vs* aHUS might be complex considering the new DIC diagnostic criteria with possible underestimation of aHUS. In addition, the quantification of ADAMTS13 activity was crucial since the overlap of clinical presentation between TTP and aHUS might delay the specific aHUS treatment with eculizumab. This might negatively impact patient recovery and the outcome of kidney function [2]. The reduction of C3 with normal C4 has been shown in aHUS due to mutations on different proteins involved in the activation and the regulation of alternative complement pathway [10, 11]. The increased C5a plasma levels support the overactivation of alternative complement pathway. In the present case this was likely triggered by the *S. pneumoniae* infection, possibly of the left maxillary sinus and lungs. Likely, the first manifestation of the infection was the hypothermia that preceded the hospital admission [10]. Interestingly, Abe et al have described a case of complement‐mediated TMA complicated with overt DIC triggered by a gram‐negative infection in which they achieved resolution of TMA using eculizumab [9].

The rare missense variant c1541T → G on *CFHR5* gene that is characterized by the aminoacid change p.Met514Arg is predicted to be pathogenic from 10 of 11 bioinformatics prediction systems (CADD 22.1). Although the c5141T → G *CFHR5* variant has been described in patients with aHUS, it is also present in the healthy control database [12]. Thus, the clinical significance of c1541T → G *CFHR5* variant is currently considered uncertain/unknown (VUS). Although VUS‐variants are not self‐sufficient to determine pathology, they might increase patient susceptibility to aHUS in association with other genetic and/or environmental factors [13]. Different studies have shown that mutations on CFHR genes are related to aHUS as well as to C3 glomerulopathy [1, 14]. Noteworthy, growing evidence indicate a role of FHR proteins as modulators/amplifiers of complement activity [1, 14]. The mutations on *CFHR5* have been reported in patients with aHUS, C3 glomerulopathy, and *S. pneumoniae* infection [1, 14]. In our case, the c1541T → G on *CFHR5* mutation might have favored an exaggerated and sustained activation of the alternative complement pathway triggered by *S. pneumoniae* infection. The excellent clinical recovery after PEX and eculizumab treatment combined with antibiotics reinforces our hypothesis.

Recently, Fakhouri and Fremeaux‐Bacchi have reported that aHUS related to rare variants on genes encoding for proteins involved in complement regulation are associated with the increased risk of relapse when eculizumab treatment is withdrawn [1]. However, in patients with aHUS in remission, discontinuation of eculizumab treatment should be considered as a concrete option, requiring a closed follow‐up. Indeed, the pros and cons for eculizumab discontinuation in this setting are in favor of discontinuation to limit disease cost and to beneficially impact patient quality of life.

## CONFLICT OF INTEREST

The authors have nothing to disclose.
